# Applying Machine Learning and SHAP Method to Identify Key Influences on Middle-School Students’ Mathematics Literacy Performance

**DOI:** 10.3390/jintelligence12100093

**Published:** 2024-09-26

**Authors:** Ying Huang, Ying Zhou, Jihe Chen, Danyan Wu

**Affiliations:** 1 School of Mathematics and Statistics, Guangxi Normal University, Guilin 541006, China; huangying199@stu.gxnu.edu.cn (Y.H.); danyanwu@stu.gxnu.edu.cn (D.W.); 2 The Faculty of Education, Southwest University, Chongqing 400715, China; chenjihe69@gmail.com

**Keywords:** machine learning, SHAP method, math literacy, key influences, PISA 2022

## Abstract

The PISA 2022 literacy assessment highlights a significant decline in math performance among most OECD countries, with the magnitude of this decline being approximately three times that of the previous round. Remarkably, Hong Kong, Macao, Taipei, Singapore, Japan, and Korea ranked in the top six among all participating countries or economies, with Taipei, Singapore, Japan, and Korea also demonstrating improved performance. Given the widespread concern about the factors influencing secondary-school students’ mathematical literacy, this paper adopts machine learning and the SHapley Additive exPlanations (SHAP) method to analyze 34,968 samples and 151 features from six East Asian education systems within the PISA 2022 dataset, aiming to pinpoint the crucial factors that affect middle-school students’ mathematical literacy. First, the XGBoost model has the highest prediction accuracy for math literacy performance. Second, 15 variables were identified as significant predictors of mathematical literacy across the student population, particularly variables such as mathematics self-efficacy (MATHEFF) and expected occupational status (BSMJ). Third, mathematics self-efficacy was determined to be the most influential factor. Fourth, the factors influencing mathematical literacy vary among individual students, including the key influencing factors, the direction (positive or negative) of their impact, and the extent of this influence. Finally, based on our findings, four recommendations are proffered to enhance the mathematical literacy performance of secondary-school students.

## 1. Introduction

The Program for International Student Assessment (PISA) is a global assessment program initiated by the Organization for Economic Co-operation and Development (OECD) to evaluate the reading, mathematics, and science literacy among 15-year-old students using standardized tests and background questionnaires (OECD and UNESCO Institute for Statistics 2003). Since 2000, PISA has been conducted every three years, with each iteration focusing on a specific domain. In 2021, the primary domain was intended to be mathematics literacy. However, due to the COVID-19 pandemic, PISA 2021 was rescheduled to 2022 and included creative thinking as an additional component of the assessment for the first time (OECD 2023b). During prior evaluations, the mean math literacy score among Organization for Economic Co-operation and Development (OECD) countries exhibited minimal variation, fluctuating within a range of 4 points. However, the results from the Program for International Student Assessment (PISA) in 2022 indicated that the average scores of 35 OECD countries, with the exception of Costa Rica and Spain, were approximately 15 points lower compared to those in 2018. This drop is more than three times as large as the fluctuations in scores of previous rounds. Notably, among the 81 participating countries and economies, the top 6 were the six East Asian education systems (Hong Kong, Macao, Taipei, Singapore, Japan, and Korea). Moreover, Taipei, Singapore, Japan, and Korea have also improved their grades. Apart from the fact that they all belong to the East Asian cultural sphere, are there any other factors that have contributed to their mathematical excellence? In other words, what are the pivotal elements that impact secondary-school students’ mathematical literacy? Moreover, mathematical literacy is an essential competency for secondary-school students in the 21st century to navigate the evolving challenges in their professional and social lives. By discerning these key influences, educators can devise targeted and practical interventions to enhance individual mathematical proficiency and better equip students for future challenges.

The PISA test employs a two-stage sampling method. The first stage uses probability proportional to size (PPS) based on school size, while the second stage involves random sampling of students within the selected schools (OECD 2003). This process generates a multilevel dataset. Consequently, existing studies have primarily utilized statistical learning methods such as multilevel linear modeling (HLM) for analyzing PISA data (Tourón et al. 2019; You et al. 2021; Lezhnina and Kismihók 2022; Bhutoria and Aljabri 2022; Hu and Yu 2021; Hu and Wang 2022). However, this study involved 34,968 samples and 152 variables (before data preprocessing), making machine learning methods more suitable for handling complex data with numerous feature dimensions and nonlinear relationships between variables (Hilbert et al. 2021). On the one hand, educational applications related to machine learning techniques, particularly performance prediction, focus more on improving model forecasting ability than on model explanation (Munir et al. 2022); on the other hand, although there is also some literature on applying machine learning methods to examine factors influencing academic performance (Lee 2022; Bernardo et al. 2022; Wang et al. 2023a; Gómez-Talal et al. 2024), they primarily explore the influencing factors for all samples, paying less attention to individual and multiple samples. Therefore, further exploration of machine learning explanations in the field of education is needed. This study identifies significant influences affecting mathematical literacy through machine learning and the SHAP method, addressing the following questions:

(1) Which machine learning model is best for predicting middle-school students’ math literacy?
(2) What are the important factors that affect mathematical literacy performance?

Moreover, previous literature has identified several variables that influence math literacy. These variables are broadly categorized into five categories in the PISA dataset: individual student, household context, school community, education systems, and macro society (Wang et al. 2023b). Since Singapore only completed the student questionnaire, school questionnaire, and creative thinking questionnaire (OECD 2023c), this article focuses on student, family, and school factors.

### 1.1. Student Factors

Student variables, including demographic characteristics, students’ psychological factors, learning opportunities, and school experiences, all exert a certain degree of impact on their mathematical literacy.

Among demographic characteristics, gender (Melkonian et al. 2019) and grade level have a significant effect on student scores. There exists a disparity between male and female students in their performance in different areas of mathematics. Male students are more dominant than females in space and shape (Liu and Wilson 2009). Nevertheless, the differences in students’ grades due to gender are not independent; rather, they are associated with the state (Zhang et al. 2022) and age (Lindberg et al. 2010). Additionally, as students progress to higher grades, their grades tend to increase (Aguayo-Téllez and Martínez-Rodríguez 2020; Spörlein and Schlueter 2018).

Regarding students’ psychology, studies have indicated that cognitive factors such as a growth mindset (Bernardo 2021) and creative thinking (de Vink et al. 2023; Sebastian and Huang 2016) can effectively raise scores. In addition to cognitive traits, non-cognitive variables such as self-concept (Pinquart and Ebeling 2020), self-efficacy (confidence) (Rodríguez et al. 2017), motivation (Pitsia et al. 2017), and positive attitudes toward learning (Bernardo 2021; Gjicali and Lipnevich 2021) are equally beneficial. In particular, self-efficacy is considered the best predictor (Stankov et al. 2012), and the impact of motivation is moderated through emotional support and self-efficacy (Skaalvik et al. 2015). However, non-cognitive features such as math anxiety (Barroso et al. 2021) may have an adverse effect. Concerning the relationship between subjective well-being and student scores, although some literature has found a positive connection (Yao et al. 2018), Bücker et al. (2018) disagree, arguing that students who are superior in math do not necessarily have a high level of well-being, and poor students do not necessarily have a lower level of well-being.

Regarding learning opportunities, the judicious utilization of Information and Communication Technology (ICT) is beneficial (Kim 2018). Students who use ICT moderately for recreation perform better than those who use it heavily (Xiao and Sun 2022). However, the availability and use of ICT both in and out of school might be detrimental (Courtney et al. 2022), while positive attitudes toward ICT could have a positive impact (Wijaya et al. 2022b). Beyond the use of ICT, students who receive early childhood education (ECE) generally have higher scores than those who do not (Giannelli and Rapallini 2016). Interestingly, an earlier start in ECE is not necessarily better. In general, pupils starting ECE at age 3 are better than those starting at 0–2, which may be related to the developmental issues of children (Laaninen et al. 2024).

Finally, features related to the school experience, such as absenteeism, truancy (Yamamura 2019; Fernández-Gutiérrez et al. 2020), and grade repetition (Salas-Velasco et al. 2021; Luschei and Jeong 2021) etc., are not conducive to promoting students’ mathematics grades.

### 1.2. Family Factors

Factors such as family socio-economic and cultural status, parental education level, and parents’ occupational status play an integral role in math literacy (Kriegbaum and Spinath 2016; Tanskanen et al. 2016). These variables are usually positively correlated with it (Sulis et al. 2020; Xiao and Sun 2021; Wang et al. 2022; Zhu et al. 2018), implying that improving these conditions contributes to student literacy. Moreover, home education resources, such as the availability of Information and Communication Technology (ICT), are also associated with it (Dockery et al. 2020; Tan 2017; Brow 2019). However, views on the impact of home ICT availability are inconsistent. Giannelli and Rapallini (2016) concluded that it is beneficial for students, while X. Hu et al. (2018) held the opposite opinion. Finally, the support provided by parents, including emotional support (Karakus et al. 2023), academic support (Levpušček and Zupančič 2009; Smith and Hausafus 1998), parental involvement (Lerner et al. 2022) and appropriate parental expectations (Daucourt et al. 2021; Tan 2017), all contribute positively to children’s achievements. In particular, parental expectation influences them through direct effects (Rodríguez et al. 2017) and indirect impacts (Pinquart and Ebeling 2020; Wijaya et al. 2022a).

### 1.3. School Factors

The school variables related to math grades primarily consist of within-classroom factors, school characteristics (Wang et al. 2023b), and teacher-related features.

For instance, larger class sizes generally promote students’ literacy in mathematics (Fung et al. 2018; Tan and Hew 2019), but different viewpoints have been found (Pivovarova and Powers 2019; Erdogdu 2022). Additionally, Denny and Oppedisano (2013) suggested that the significance of class size is linked to the method of analysis used. Apart from class size, the number of teachers is also an important factor in the improvement of math accomplishments. A modest increase in the student-teacher ratio is generally favorable (Gamazo and Martínez-Abad 2020; Erdogdu 2022), while Bokhove and Hampden-Thompson (2022), on the other hand, concluded that the student-teacher ratio does not have a significant impact.

Regarding school characteristics, it has been demonstrated that mathematical literacy is strongly associated with school Information and Communication Technology (ICT) usage (Juhaňák et al. 2018; Wang and Wang 2023), and it is considered beneficial (Hu et al. 2018), even though Eickelmann et al. (2012) and Posso (2016) hold the opposite view. Generally, students in urban schools are more literate than those in rural areas (Dockery et al. 2020; Fernández-Gutiérrez et al. 2020), although Bokhove and Hampden-Thompson (2022) hold a different stance, while Bhutoria and Aljabri (2022) found that its impact is not significant. As for school size, Gümüş et al. (2022) argued that larger schools are better, while Erdogdu (2022) disagreed. In addition, a strong sense of school belonging (Evans and Field 2020) and moderate school competition (Rudolf and Lee 2023) also relate to it. Finally, school bullying is generally considered to harm mathematical achievements (Konishi et al. 2010), and this effect acts indirectly by reducing students’ subjective well-being in school.

Research indicates that students’ perception of teacher support (Ma et al. 2021) and access to teacher support (Mammadov and Schroeder 2023), as well as feedback (Forsythe and Johnson 2017), have a positive effect on their literacy; however, the use of written teacher feedback may be disadvantageous, suggesting that the effect of teacher feedback on mathematical literacy is related to the form of feedback (Selvaraj et al. 2021). Furthermore, high-quality teacher-student relationships are thought to enhance students’ mathematical literacy, and this effect is indirectly achieved by increasing teachers’ self-efficacy (Hajovsky et al. 2020). Regarding cognitive activation (You et al. 2021), Lee (2021) found that cognitive activation was positively associated with students’ mathematics results in Confucian areas. At the same time, it was also noted that the effect was related to the frequency (Caro et al. 2016) and the activity type of cognitive activation (Tourón et al. 2019).

Finally, the purpose of this study is to reveal the important factors affecting mathematics literacy by considering numerous features and highlighting the explanation ability of machine learning approaches in the field of pedagogy. At the same time, the findings of global explanation also provide data support for the upgrading of the education system, and the results of local explanation assist in the development of personalized teaching in the context of artificial intelligence.

## 2. Materials and Methods

### 2.1. The Data Sources and Processing

#### 2.1.1. Data Sources

PISA 2022 employs 10 plausible values (PVs) to represent each student’s mathematical literacy, aiming to assess their abilities more accurately. Additionally, the PISA data analysis manual states that “the use of one plausible value or five plausible values does not really have a substantial impact on a large sample.” (OECD 2009) Therefore, the first plausible value (PV1MATH) was selected as each student’s math literacy score. The dataset was obtained from the PISA 2022 of six East Asian education systems (Hong Kong, Macao, Taipei, Singapore, Japan, and Korea), with a total of 34,968 middle-school student samples. From it, 151 characteristics related to individual students (77), household backgrounds (17), and school community (including teachers) (57) were selected as input variables, and PV1MATH was used as an output variable.

#### 2.1.2. Data Processing

**Figure 1:**
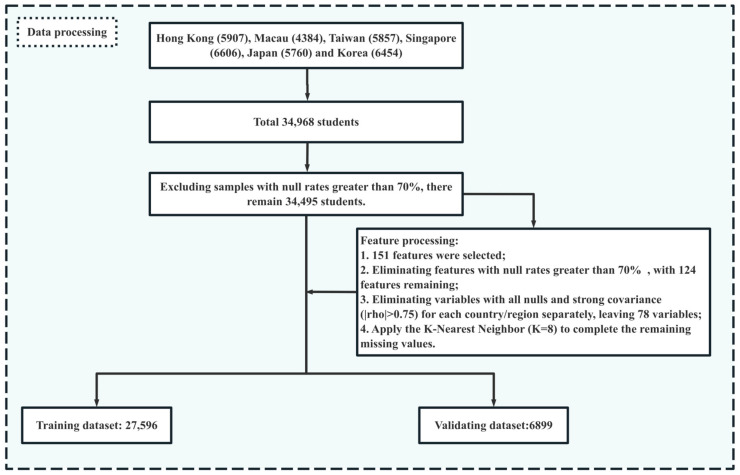
Flowchart of data processing.

Data processing mainly encompasses the steps of selecting features and handling missing values (Figure 1), and the detailed process is as follows. First, samples with a missing rate greater than 70% are excluded. After excluding samples with null values exceeding 70% from the selected 34,968 samples, 34,495 samples remained. Second, features are selected. This study chooses 151 characteristics related to student factors (77), household factors (17), and school factors (57) as independent variables. Among them, student variables contain factors such as grade, math anxiety, and self-efficacy; family features include indicators such as ESCS, family support, and parental expectations; and school variables are related to the sense of belonging, teacher feedback and support, class size, etc. Third, features with a missing rate of more than 70% are eliminated. After removing features with nulls of more than 70% from the selected 151 input variables, 124 features are retained. Fourth, variables with all missing data for each country/region are excluded separately. Some countries/regions in the six East Asian education systems did not participate in the optional questionnaire. For example, Japan, Korea, Singapore, and Taipei did not engage in the well-being questionnaire (OECD 2023a), and the data for them are missing. Thus, this study excludes the characteristics of all missing data for each of them and retains 78 variables. Fifth, variables with moderate/strong multicollinearity are removed. Some of the included variables may have collinearity. For example, ESCS is derived from the highest parental occupation status (HISEI), highest education of parents in years (PAREDINT), and home possessions (HOMEPOS). To avoid covariance among the features reducing the prediction performance of methods, six variables (ICTWKDY, HOMEPOS, PAREDINT, ICTAVSCH, ICTAVHOM, and MCLSIZE) with moderate covariance are excluded, ultimately leaving 72 independent variables (see Table S2). Sixth, the KNN algorithm (K = 8) (Bernardo et al. 2023) is used to complete the missing values. Finally, a feature dataset consisting of 34,495 samples and 72 variables is obtained. Seventh, the dataset is divided into the training and validating datasets in a ratio of 80:20. In this case, the sample size of the training dataset and validating dataset are 27,596 and 6,899, respectively.

### 2.2. Methods

#### 2.2.1. Machine Learning Methods

Reference is made to the models commonly used in performance prediction (Munir et al. 2022). Multiple Linear Regression (MLR) (Zhou 2021), Support Vector Regression (SVR) (Li 2023), Decision Tree (DT) (Zhou 2021), Random Forest (RF) (Segal 2004), and XGBoost (Acıslı-Celik and Yesilkanat 2023) were included. Next, GridSearchCV was applied to exhaustively enumerate the given combinations of hyperparameters to determine a set of optimal hyperparameters for the five algorithms. Then, with the help of metrics such as Mean Absolute Percentage Error (MAPE) and Pearson’s Correlation Coefficient (PCCs), the prediction ability of the five optimal models in PISA 2022 mathematical literacy of Hong Kong, Macao, Taipei, Singapore, Japan, and Korea was assessed. After comparing their prediction performance, it was found that the XGBoost model was considered the best.

(1) Multiple linear regression (MLR)

Multiple linear regression is a predictive modeling technique. It employs curves or surfaces to fit some known data points, with the objective of minimizing the difference in distance from the curve or surface $f(x)=\omega^{T}x+b$ to the data points. For a given dataset $D={\left\{(x_{i},\;y_{i})\right\}}_{i=1}^{n}$, where $x_{i}\in R^{n}$, the loss function can be defined as follows:

(1) $$L(\omega ,\;b)=\frac{1}{n}\sum_{i=1}^{n}{\left(\omega x_{i}+b-y_{i}\right)}^{2}\;$$

The current task is transformed into minimization L(ω, b), i.e.,:

(2) $$\underset{\omega ,b}{\mathrm{arg min}}\sum_{i=1}^{n}{\left(\omega x_{i}+b-y_{i}\right)}^{2}$$

Then, the gradient descent method is employed to minimize the objective function (loss function).

(2) Support vector machine regression (SVR)

Given a dataset $D={\left\{(x_{i},\;y_{i})\right\}}_{i=1}^{n},\;x_{i}\epsilon R^{n}$, we aim to learn a regression model $f(x)=\omega^{T}x+b$ that is as close as possible to the data points y, here ω and *b* are the model parameters. In the support vector machine regression algorithm, the loss function is computed only when the absolute value of the difference between f(x) and *y* is more than the error ε. Therefore, the support vector machine regression problem can be written in the following form:

(3) $$\underset{\omega ,b}{\min}\;\;\frac{1}{2}{\left\|\omega \right\|}^{2}+C\sum_{i=1}^{n}l_{\varepsilon }(f(x_{i})-y_{i})$$

where *C* is a regularization constant, $l_{\varepsilon }=\left\{\begin{array}{c} 0,\;if\;\left|z\right|<\varepsilon \\ \left|z\right|-\varepsilon ,\;otherwise \end{array}\right.$, and by introducing slack variables $\xi_{i}$ and ${\hat{\xi }}_{i}$, the above problem can be written as:

(4) $$\underset{\omega ,b,\;\xi_{i},\;{\hat{\xi }}_{i}\;}{\min}\;\;\frac{1}{2}{\left\|\omega \right\|}^{2}+C\sum_{i=1}^{n}\left(\xi_{i}+{\hat{\xi }}_{i}\right)\;s.t.\;f(x_{i})-y_{i}\leq \varepsilon +\xi_{i}\;y_{i}-f(x_{i})\leq \varepsilon +{\hat{\xi }}_{i}\;\xi_{i}\geq 0,\;{\hat{\xi }}_{i}\geq 0,\;i=1,2,\cdots n$$

Then, the Lagrange multipliers are introduced to obtain the Lagrange function. The optimal solution to the original problem is obtained by solving its dual problem.

(3) Decision tree (DT)

A regression tree has continuous predictive values, and the average of all samples at a leaf node is usually taken as the prediction value for that node. Its generation is a process of recursively constructing a binary tree. For a given dataset $D={\left\{(x_{i},\;y_{i})\right\}}_{i=1}^{n},\;x_{i}\epsilon R^{n}$, the *j*-th variable $x_{j}$ and its value *s* are chosen as the cutoff variable and cutoff point, and two regions are defined:

(5) $$R_{1}(j,s)=\left\{x|x_{j}\leq s\right\},\;R_{2}(j,s)=\left\{x|x_{j}>s\right\}\;$$

Then, find the optimal cutoff variable *j* and cutoff point *s*. Specifically, solve for them.

(6) $$\underset{j,s}{\min}\left[\underset{c_{1}}{\min}\sum_{x_{i}\in R_{1}(j,\;s)}{\left(y_{i}-c_{1}\right)}^{2}+\underset{c_{2}}{\min}\sum_{x_{i}\in R_{2}(j,\;s)}{\left(y_{i}-c_{2}\right)}^{2}\right]$$

The optimal cut-point *s* can be found for a fixed input variable *j*. The optimal cut-point *s* can be found for a fixed input variable *j*.

(7) $${\hat{c}}_{1}=ave\left(y_{i}|x_{i}\in R_{1}(j,s)\right)\;{\hat{c}}_{2}=ave\left(y_{i}|x_{i}\in R_{2}(j,s)\right)$$

Iterate over all input variables to find the optimal cutoff variable *j*, forming a pair (*j, s*). Sequentially, the space is divided into two regions. Then, repeat the above division process for each region until the stopping condition is satisfied.

(4) Random forest (RF)

**Figure 2:**
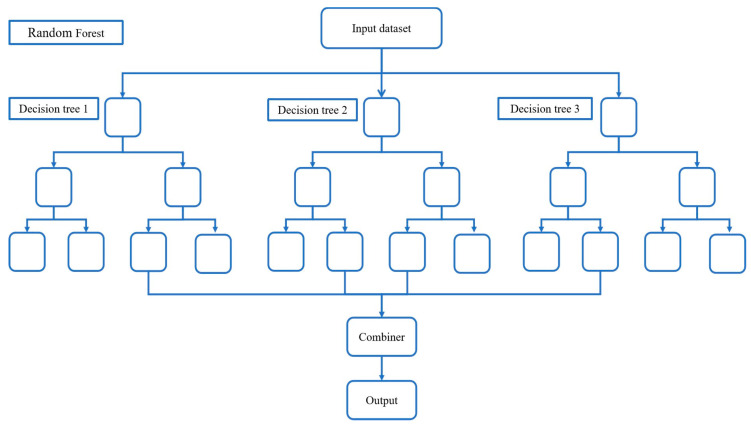
A random forest consisting of three decision trees.

Random forest is a combination of bagging and decision trees (Figure 2). The main idea is to train several decision tree models (learners) separately and then let all the learners decide the output of the test samples with an equal voting mechanism. In addition, the training samples of each learner are randomly selected, i.e., an equal number of data are randomly drawn from the original training dataset with replacement as the training samples; and when building the decision tree, a portion of the features is randomly selected from the features to build the decision tree. For the regression problem, Random Forest uses the CART regression tree as an individual learner and adopts the mean of the outputs of all CART regression trees as the final prediction result.

(5) XGBoost

Extreme Gradient Boosting (XGBoost) was proposed by (T. Chen and Guestrin 2016). Although the popularity of neural networks has increased dramatically in recent years, XGBoost still demonstrates unparalleled and unique advantages in the face of limited training samples, short training cycles, or inexperience in tuning. It is also better at handling structured tabular data and model explanations. XGBoost is a supervised learning algorithm based on Gradient Tree Boosting, which can solve problems such as classification and regression. First, it assigns an initial weight $\omega_{i}\;\left(i=1,2,\cdots ,n\right)$ to each training sample. In each iteration, XGBoost trains a decision tree. Second, it adjusts the weights of training samples according to the forecast ability of decision trees. Then, it trains the next round of the weak learner (base learner) on the adjusted dataset and repeats the above process until it reaches the preset stopping condition. Finally, a strong learner is constructed by combining all weak learners through a specific combining strategy.

Assuming that the training dataset is $D=\left\{\left(x_{i},y_{i}\right)\right\},x_{i}\in R^{m},y_{i}\in R$, the model contains *K* decision trees. Then, the XGBoost model is defined as follows:

(8) $${\;\hat{y}}_{i}=F_{K}\left(x_{i}\right)=F_{K-1}\left(x_{i}\right)+f_{K}\left(x_{i}\right)$$

$f_{K}\left(x_{i}\right)$ denotes the *K*-th decision tree, and $F_{K-1}\left(x_{i}\right)$ is the first *K*-1 base learner that has completed training and is fixed. Its objective function is:

(9) $$Obj=\sum_{i=1}^{n}L\left(y_{i},{\hat{y}}_{i}\right)\;+\sum_{k=1}^{K}\Omega \left(f_{k}\right)\;\;\;\begin{array}{c} =\sum_{i=1}^{n}L\left[y_{i},F_{K-1}\left(x_{i}\right)+f_{K}\left(x_{i}\right)\right]+\sum_{k=1}^{K}\Omega \left(f_{k}\right) \end{array}$$

$L\left(y_{i},\;{\hat{y}}_{i}\right)$ represents the loss function, which is used to evaluate the error between the actual value and the model prediction, and $\Omega \left(f\right)=\gamma T+\frac{1}{2}\lambda {\left\|\omega \right\|}^{2}$ is the $L_{2}$ regular term; *T* is the leaf nodes of tree f; ω is a vector consisting of the output values of all leaf nodes (prediction values); γ and λ are the hyperparameters. For a given sample $x_{p}$, its forecast is obtained by summing the outputs of the *K* decision trees.

#### 2.2.2. SHAP Method

Considering the extant literature on predicting student accomplishments based on machine learning methods, most of them focus on the capability of model forecast (Munir et al. 2022) and lack sufficient attention to the explanation of model outputs. The article applies the SHAP method to explain how different factors affect the generation of forecast values (mathematical literacy). SHapley Additive exPlanation (SHAP) is a method for explaining machine learning models based on cooperative game theory. It explains the output of the model by calculating how much each feature contributes to the prediction value. The formula is shown below:

(10) $$\begin{array}{c} {\hat{y}}_{i}={\hat{y}}_{base}+{\hat{y}}_{i1}+{\hat{y}}_{i2}+\cdots +{\hat{y}}_{im} \end{array}$$

${\hat{y}}_{i}$ represents the prediction value of the *i*-th sample and ${\hat{y}}_{base}$ is the average of the predicted values of all samples, and ${\hat{y}}_{ij}$ is the SHAP value of the *j*-th feature in the *i*-th sample, which is used to reflect the extent to which feature *j* contributes to the model output of sample *i*. ${\hat{y}}_{ij}>0$ means that feature *j* has a positive effect on the predicted value of sample *i*; ${\hat{y}}_{ij}<0$ denotes that feature *j* hurts sample *i* and m is the total number of features. There are several variants of the SHAP method, for example, TreeSHAP (Lundberg and Lee 2017), which is particularly suitable for tree-based methods such as XGBoost. In addition, the TreeSHAP method is widely popular for its fast computational speed and high accuracy. Therefore, this paper primarily explains the output results of the XGBoost model with the help of the TreeSHAP method.

## 3. Results

### 3.1. Model Training and Prediction Ability

**Figure 3:**
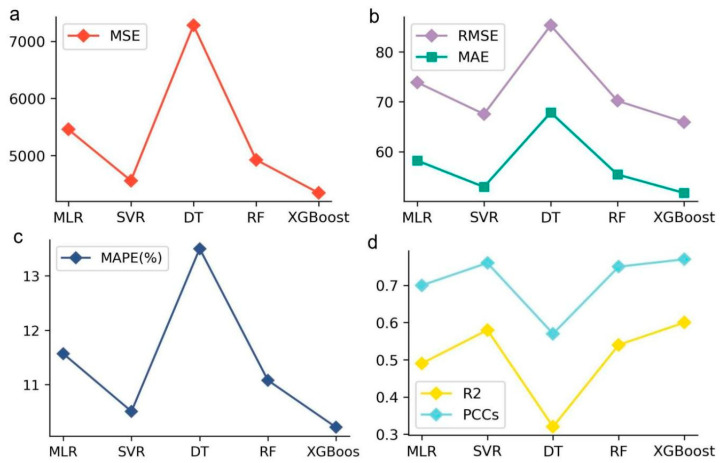
Model evaluation metrics for different methods on the validating dataset. (**a**) Mean Square Error (MSE). (**b**) Root Mean Square Error (RMSE) and Mean Absolute Error (MAE). (**c**) Mean Absolute Percentage Error (MAPE). (**d**) Coefficient of Determination $\left(R^{2}\right)$ and Pearson Correlation Coefficients (PCCs).

To make the model have a better prediction performance (accuracy), all combinations of the given hyperparameters were exhaustively enumerated using GridSearchCV (5-fold cross-validation by default), and a set of optimal hyperparameters was obtained for Multiple Linear Regression (MLR), Support Vector Regression (SVR), Decision Tree (DT), Random Forest (RF), and XGBoost, respectively (See Table 1). Then, the forecast capability of the optimal hyperparameter models on the validating dataset was compared (see Table 2 and Figure 3). The model evaluation metrics include Mean Square Error (MSE), Root Mean Square Error (RMSE), Mean Absolute Error (MAE), Mean Absolute Percentage Error (MAPE), Coefficient of Determination $\left(R^{2}\right)$ and Pearson Correlation Coefficients (PCCs). In particular, PCCs are used to measure the degree of linear correlation between model outputs and actual values.

In general, when the MAPE is less than 10%, the model is considered to have good predictive ability, and when the MAPE is between 10% to 20%, the predictive effect of the model is considered to be acceptable. As can be seen from Table 2 and Figure 3c, the MAPE of all the models on the validating dataset is between 10% to 15%, which implies that the prediction ability of these models is acceptable. In particular, the evaluation indicators of the XGBoost model (MSE = 4344.98, RMSE = 65.92, MAE = 51.72, and MAPE = 10.21%) are lower than those of MLR, SVR, DT, and RF. Moreover, its coefficient of determination ($R^{2}=0.60$), and Pearson’s correlation coefficients (PCCs = 0.77) are also superior to the other four models. In addition, this study also gives the prediction performance of the XGBoost model on the Hong Kong, Korea, US, and Spain datasets, and the results show that the prediction ability is within the acceptable range for all regions except Spain (see Figures S1–S4, Table S2). Based on the above analysis, it can be seen that the XGBoost model is the most superior regression model for predicting the mathematical literacy of the PISA 2022 in Hong Kong, Macao, Taipei, Singapore, Japan, and Korea.

### 3.2. Model Explanation

The SHAP method provides two types of explanations, including global model explanation and local model explanation. The global explanation aims to assess the contribution of each feature to the predicted outcome for all samples. The SHAP summary bar plot determines the importance of the feature to the prediction value by calculating the average of the absolute values of the SHAP values of all samples for each feature. The SHAP summary dot plot presents the SHAP values for each sample. In addition, the SHAP partial dependence plot gives the specific contribution of single features to the model output. The local explanation is concerned with evaluating the contribution of each feature to the prediction results for a specific individual sample and multiple samples.

#### 3.2.1. The Number of Key Influencing Factors

**Figure 4:**
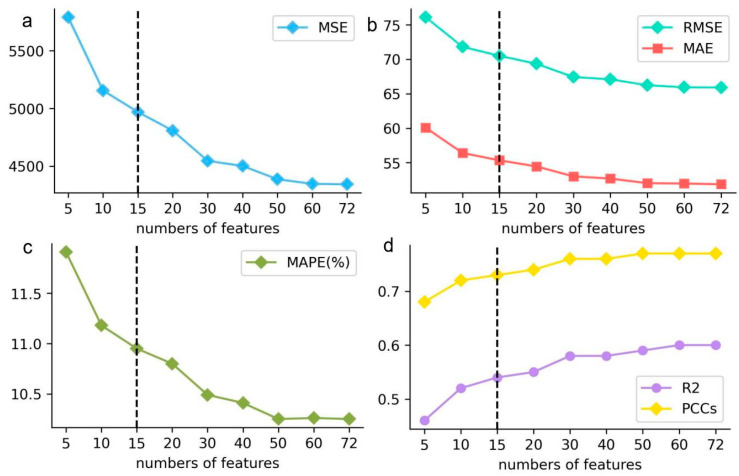
Prediction ability of XGBoost with a different number of features.

In feature importance analysis, the top 10 (Bernardo et al. 2022), top 15 (Bernardo et al. 2023), and top 20 (Chen et al. 2021) variables are considered to be important influences on students’ literacy. In this study, after identifying XGBoost as the optimal model for predicting math literacy in middle-school students, the SHAP method was employed to evaluate the contribution of each variable to the predictions (math literacy). Subsequently, by comparing the prediction performance (using a validating dataset) of the XGBoost model with varying numbers of features (top 5, top 10, top 15, top 20, top 30, top 40, top 50, top 60, and the full set of features (72)), the key influence numbers were determined. From Table 3 and Figure 4, it can be seen that, on the one hand, the prediction accuracy of the 15-feature model was within an acceptable range (MAPE = 10.95%); on the other hand, the Mean Absolute Percentage Error (MAPE = 10.95%) and Pearson correlation coefficients (PCCs = 0.73) of the 15-feature model were slightly inferior compared to those of the 72-feature model (MAPE = 10.25%, PCCs = 0.77). In addition, the top 15 variables, in terms of feature importance, were able to explain the influence of the educational system on students’ math literacy (Bernardo et al. 2023). Therefore, in this paper, the number of key factors affecting math literacy was set at 15.

#### 3.2.2. All Samples Analysis

**Figure 5:**
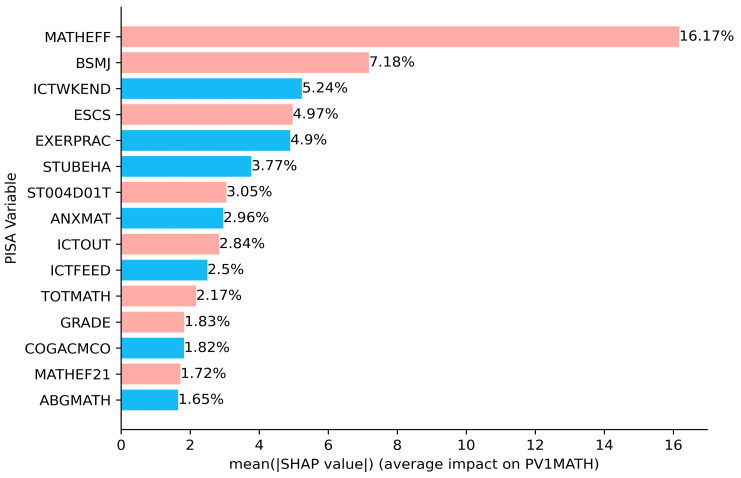
SHAP summary bar plot.

**Figure 6:**
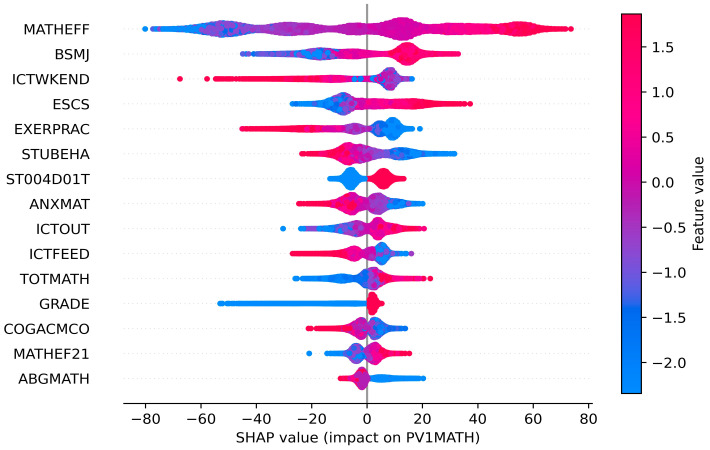
SHAP summary dot plot.

**Figure 7:**
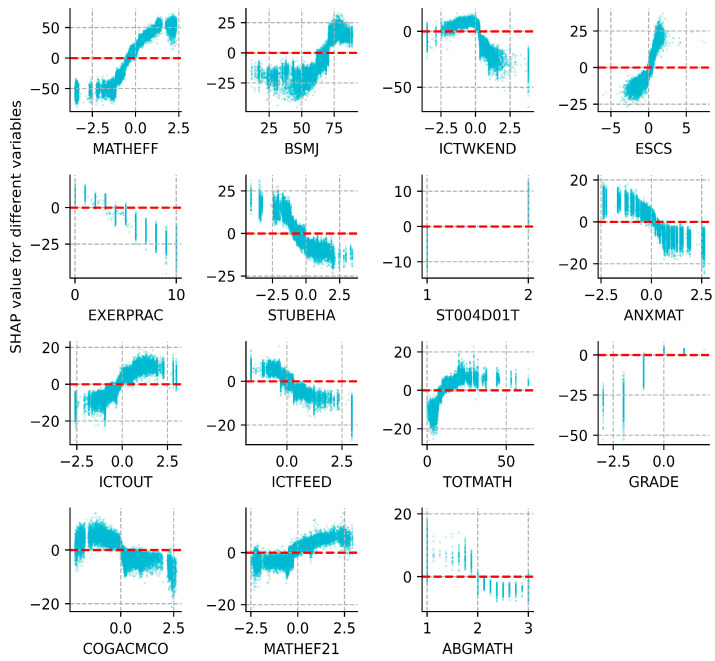
SHAP partial dependence plot.

The SHAP summary bar plot ranks the variables by the mean of the absolute values of all students’ SHAP values. On the one hand, the color of the bar represents whether the independent variable positively or negatively affects the forecasts, with red showing a positive contribution and blue the negative. For example, mathematical self-efficacy (MATHEFF) and mathematical anxiety (ANXMAT) have positive and negative impacts on mathematical literacy, respectively (Figure 5). On the other hand, the length of the bar indicates the importance of the feature, with a longer bar indicating that the feature contributes more to the model outputs. For example, mathematical self-efficacy (MATHEFF) has the greatest contribution (Figure 5). The dot plot shows the SHAP value for every sample (Figure 6). Math scores increase as the SHAP value of the feature increases. Each student has one dot on the line of each feature, and these dots are stacked vertically to show the density. The color of the dots changing from blue to red indicates the actual value of the variable changes from small to large. In addition, the SHAP partial dependence plot (Figure 7) gives the contribution of the signal input variable to predictions. A dot represents one student. The *x*-axis represents the actual value of the independent variable, and the *y*-axis is the SHAP value of that variable. For example, both MATHEFF > −0.5 and ANXMAT < 0 tend to increase math accomplishments, while MATHEFF < −0.5 and ANXMAT > 0 tend to decrease. Finally, the key factors influencing PISA 2022 mathematics literacy in Hong Kong, Macao, Taipei, Singapore, Japan, and Korea and their contributions are presented in detail below.

1. Key influencing factors
  (1) Student factors

Student factors related to mathematical literacy include demographic variables such as student gender (ST004D01T) and grade level (GRADE), as well as psychological factors such as mathematical self-efficacy (MATHEFF, MATHEF21), expected occupational status (BSMJ) and math anxiety (ANXMAT). In addition, variables related to learning opportunities are the frequency of using Information and Communication Technology (ICT) for leisure activities on weekends (ICTWKEND), participation in physical activity before and after school (EXERPRAC), the frequency of receiving support and feedback from teachers and classmates with the help of ICT (ICTFEED), and use of ICT outside the classroom for learning activities (ICTOUT). Meanwhile, the school experience focuses on factors that disrupt the school climate, such as truancy and alcoholism (STUBEHA).

ST004D01T refers to student gender. On the one hand, there is a positive contribution to math literacy (Figure 5); on the other hand, when ST004D01T = 1, the SHAP value is below 0, and the individual’s prediction value will be lower than the average of all the samples without considering the effects of other features. In contrast, when ST004D01T = 2, the prediction of math literacy is greater than the mean of all samples (Figure 5). GRADE indicates comparison to modal grade in the country. It exhibits a positive relationship with their accomplishments, i.e., the higher the grade level the student is in, the better the math accomplishments generally are. In particular, GRADE > 0 tends to increase students’ scores; conversely, it inclines to reduce them (Figure 7).

MATHEFF represents mathematics self-efficacy in doing a range of formal and applied mathematics tasks. MATHEF21 denotes mathematical reasoning and 21st-century mathematics tasks. They show a positive relationship with students’ math scores. Meanwhile, MATHEFF > 0 and MATHEF21 > 0 are both favorable to enhance the prediction. BSMJ refers to the students’ expected occupational status, and higher scores on this variable indicate higher levels of a student’s expected occupational status. When BSMJ > 70, most of the students’ SHAP values are larger than 0, i.e., while the effect of other independent variables is not taken into account, there is an increase in math literacy. ANXMAT means mathematics anxiety, and it shows a negative effect. At the same time, ANXMAT < 0 tends to boost students’ accomplishments.

The frequency of ICT activity during a weekend day (ICTWKEND), the days of exercising or practicing a sport before or after school (EXERPRAC), and ICTFEED, which indicates the support or feedback from teachers and other students via ICT, all show a significant negative relationship with pupils’ attainment. This indicates that as these variables increase, their accomplishment declines. However, the use of ICT for school activities outside of the classroom (ICTOUT) generates different influences, which means an appropriate increase in the use of ICT for school-related activities contributes to student grades. Furthermore, ICTWKEND > 0, EXERPRAC > 4, ICTFEED > 0, and ICTOUT < 0 all tend to boost the scores.

STUBEHA means student-related factors affecting school climate, such as student truancy and student use of alcohol or illegal drugs, and it is harmful to their math accomplishments. Alternatively, when STUBEHA < -0.5, most samples have SHAP values less than 0, which suggests that the math grades rise.

  (2) Family factors

The index of economic, social, and cultural status (ESCS) is based on three indicators: highest parental occupation status (HISEI), highest education of parents in years (PAREDINT), and home possessions (HOMEPOS). Typically, math literacy shows an upward trend as ESCS improves. And if ECSE < 0, it would tend to lower the scores.

  (3) School factors

They can be broadly grouped into within-classroom factors (TOTMATH), school characteristics (ABGMATH), and teacher factors (COGACMCO). The total number of mathematics teachers at school (TOTMATH) has a positive association with the predicted scores, and TOTMATH < 0 tends to reduce their literacy. However, the ability grouping for mathematics classes (ABGMATH) is harmful, revealing that in mathematics classes, blindly teaching students in groups according to their abilities or grades does not always foster an increase in math accomplishments. As for teacher factors, cognitive activation in mathematics (COGACMCO) does not act as a positive influence, as can be seen in Figure 7. In other words, teachers’ moderate use of cognitive activation activities is beneficial to the individual’s success, while excessive or abusive use of cognitive activation activities may lead to a decrease.

2. Contribution of key influencing factors

The extent to which each significant factor contributed to the PISA 2022 math literacy in Hong Kong, Macao, Taipei, Singapore, Japan, and Korea, in descending order, is as follows: MATHEFF (16.17%), MATHEF21 (1.72%) > BSMJ (7.18%) > ESCS (4.97%) > ICTWKEND (5.24%) > EXERPRAC (4.9%) > STUBEHA (3.77%) > ST004D01T (3.05%) > ANXMAT (2.96%) > ICTOUT (2.84%) > ICTFEED (2.5%) > TOTMATH (2.17%) > GRADE (1.83%) > COGACMCO (1.82%) > ABGMATH (1.65%) (see Figure 5). Mathematics self-efficacy in expressing and applying math to solve practical problems in mathematics had the most significant effect on mathematics literacy, followed by expected occupational status, then the use of ICT for recreational activities on weekends, and so on. Lastly, the school’s ability grouping for mathematics classes had the least impact.

#### 3.2.3. Specific and Multiple Sample Analysis

**Figure 8:**
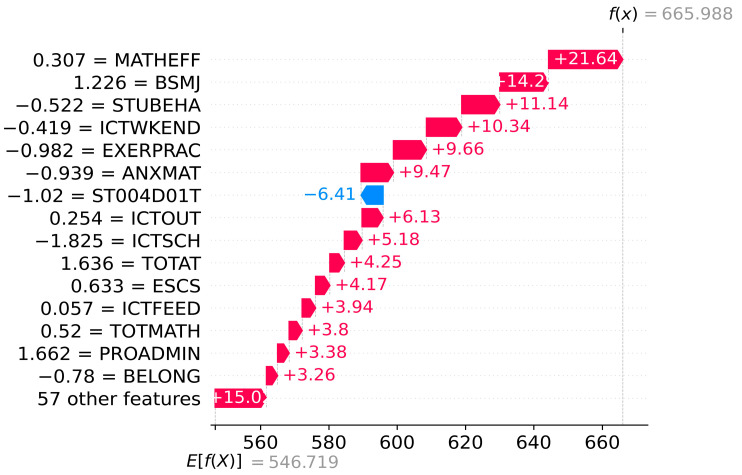
Waterfall plot for student A.

**Figure 9:**
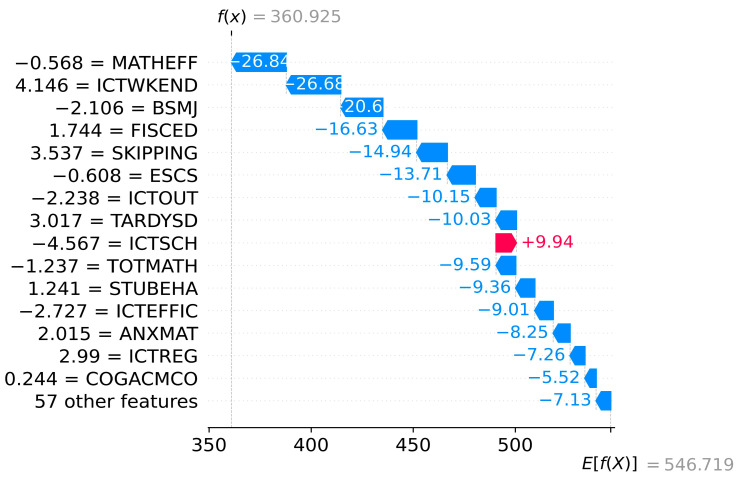
Waterfall plot for student B.

Beyond the global model explanation, SHAP analysis also provides a local model explanation for single or multiple students. Figure 8 and Figure 9 show the localized bar graphs of a top-performing student A $\left(PV1MATH\geq 607\right)$ and a low-performing student B $\left(PV1MATH<420\right)$, respectively. $f\left(x\right)$ is the prediction value, and $E\left[f\left(x\right)\right]$ presents the mean of the model outputs of all samples $\left(E\left[f\left(x\right)\right]=546.719\right)$. The lengths of the red and blue bars indicate the extent of the contribution of each variable to the forecasts, where the red bar shows a positive contribution, and the blue bar shows the opposite. Thus, the forecasts of mathematical literacy of student A and student B are 665.988 and 360.925. The variables ESCS, BSMJ, and STUBEHA increase the scores of student A by 21.64, 14.29, and 11.14, respectively, from the mean scores of all samples. ICTSCH increases the literacy of student B by 9.94. Similarly, ST004D01T results in a decrease of 6.41 for student A compared to the mean score ($E\left[f\left(x\right)\right]$); MATHEFF, ICTWKEND, and BSMJ lead to the forecast of student B to decline by 26.84, 26.68, and 20.63, respectively.

**Figure 10:**
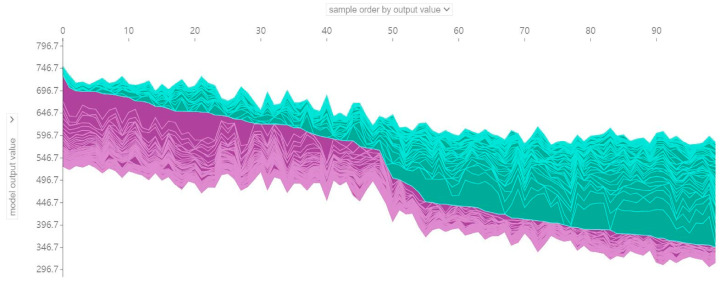
Force plot for the 100 students.

In the Force plot, the *x*-axis indicates each student, while the *y*-axis represents the SHAP value of each feature, i.e., the extent to which it contributes to the prediction value. The features marked in purple are all factors that boost mathematic accomplishments, while the variables marked in green all decrease. Figure 10 presents a force plot consisting of SHAP values of 100 samples, where the first 50 samples belong to top-performing students in mathematics and the last 50 are low-performing students. It can be seen from left to right that the number of features that enhance mathematical literacy gradually decreases.

## 4. Discussion

(1) XGBoost is the best model for predicting the mathematical literacy of middle-school students.

Several studies have utilized Support Vector Machines (SVM) (Gorostiaga and Rojo-Álvarez 2016) and Random Forest (RF) (Bernardo et al. 2022) to predict students’ mathematics literacy. This paper compares the predicting capability of five algorithms, including SVM, RF, and XGBoost models in PISA 2022 mathematics literacy scores in Hong Kong, Macao, Taipei, Singapore, Japan, and Korea, and finds that XGBoost is the optimal model.

(2) A total of 15 variables, including math self-efficacy, are key influences on math literacy.

The global model explanation identifies 15 factors that are important in determining the math literacy of the whole sample, including math self-efficacy (MATHEFF, MATHEF21), expected occupational status (BSMJ), frequency of participation in leisure activities via Information and Communication Technology (ICT) (ICTWKEND), and so on. Among these variables, eight variables, such as math self-efficacy, expected occupational status, and family’s socio-economic and cultural status (ESCS), contribute positively to mathematical literacy, while seven variables, such as the use of ICT for leisure activities (ICTWKEND), participation in physical activity (EXERPRAC), and math anxiety (ANXMAT) have the opposite contribution. Moreover, these variables are primarily related to student, family, and school factors and are dominated by individual student factors.

In terms of student factors, the larger the value of gender (ST004D01T), the better their attainment, which signifies that boys outperform girls in math literacy accomplishments overall (Liu and Wilson 2009; Gevrek et al. 2020). The contribution of grades to it is positive, which says that students tend to perform better as they move up the grade scale (Aguayo-Téllez and Martínez-Rodríguez 2020). The reason for this is that students in higher grades have a broader base of mathematical knowledge than students in lower grades, and breadth of knowledge tends to show a positive link to their grades (Barnard-Brak et al. 2018).

Both MATHEFF and MATHEF21 are relevant to student self-efficacy. In general, students with stronger self-efficacy are favored, and this finding is consistent with the results of X. Wang et al. (2024), Brow (2019), and Zhao and Ding (2019). Furthermore, the higher the expected occupation status (BSMJ), the more excellent their math outcomes (Bernardo et al. 2022; Tourón et al. 2018). Also, the contribution of BSMJ is followed by MATHEFF, which demonstrates that it is critical to encourage students to engage in activities that foster intrinsic motivation to raise the level of educational and occupational aspirations (Michael and Kyriakides 2023). Nevertheless, students with increased levels of anxiety in math (ANXMAT) perform worse (Barroso et al. 2021; Fan et al. 2019; Cheung 2017). When the value of ANXMAT is more than 0, it tends to a drop in the model outcome, which implies that excessive math anxiety is detrimental.

Regarding learning opportunities, the frequency of ICT activity on weekends (ICTWKEND) is not conducive. This phenomenon is explained by the fact that students who spend too much time on online social networks significantly negatively affect other domains of learning (Cheema and Zhang 2013; Posso 2016). On the contrary, the use of ICT for school activities outside of the classroom (ICTOUT) is beneficial since using ICT for learning relevant activities is useful to their accomplishments (Skryabin et al. 2015). Hence, there is still a need for further exploration to reveal the connection between ICT and pupils’ performance. ICTFEED describes the frequency of students’ use of ICT in various activities related to support or feedback, which may generate a disadvantageous effect. Several studies have shown that teacher support facilitates students’ academic performance (Ma et al. 2021; Mammadov and Schroeder 2023), and students’ positive attitudes toward peer and teacher feedback also contribute to it (Forsythe and Johnson 2017). Yet, Selvaraj et al. (2021) note that although teacher feedback can contribute to it, the form of the feedback, especially written form, may adversely reflect on or be a barrier to student learning. Consequently, the links between the form of feedback and students’ performance in mathematical literacy remain to be thoroughly analyzed. Exercising or practicing a sport before or after school (EXERPRAC) yields a slight negative effect, which may be related to the fact that physical activity reduces the time available for study (Aksoy and Link 2000).

Lastly, regarding school experiences, student-related factors affecting school climate (STUBEHA) are hazardous to student grades, which suggests that curbing undesirable behaviors such as truancy is beneficial (Fernández-Gutiérrez et al. 2020; Yamamura 2019; Sälzer and Heine 2016).

Family factors like the index of economic, social, and cultural status (ESCS) contribute favorably, and enhancing it helps to upgrade students’ mathematics grades (Sulis et al. 2020; Xiao and Sun 2021; Wang et al. 2022; Zhu et al. 2018), as more educated parents can provide more assistance in terms of occupational status and economic resources to their children than less educated (Useem 1992).

As for school factors, TOTMATH expresses the total number of mathematics teachers at school. At TOTMATH < 0, their mathematical grades will decline due to a shortage of teachers is harmful to students’ mathematics study (Bokhove and Hampden-Thompson 2022). In the alternative, some countries, to improve the scores of underperforming students, match them into smaller classrooms for learning (Wang et al. 2023b). Second, ABGMATH reflects the extent to which school mathematics programs are adapted to students of different ability levels. Although Slavin (1987) found that grouping students according to their ability or grades was useful in promoting their math scores, the research by Betts and Shkolnik (2000) stated that school policies that grouped students by ability nearly contributed positively to student literacy, while in heterogeneous classes, middle and poor students tended to perform better in math than their peers in same-ability classes (Linchevski and Kutscher 1998), which may help to explain the result that ABGMATH contributed negatively to math scores. Regarding cognitive activation in mathematics (COGACMC), unlike the findings of Lee (2021), who reports that cognitive activation shows a positive relationship with students’ literacy in Confucian areas, this essay discovers that COGACMC is damaging to their grades, and this gap may link to the frequency (Caro et al. 2016) and type (Tourón et al. 2019) of cognitive activation activities implemented by teachers.

(3) Math self-efficacy is the most significant influence on math literacy.

Among the important factors affecting students’ literacy in mathematics, self-efficacy in applying mathematics to solve real-world problems (16.17%) and self-efficacy in engaging in mathematical reasoning and solving 21st-century mathematical problems (1.72%) contribute the most. This is followed by expected occupation status (7.18%), and finally, ability grouping for mathematics classes (1.65%), i.e., math self-efficacy has the most significant impact on mathematical literacy (You et al. 2021). Additionally, mathematical self-efficacy makes a positive contribution to math literacy. Combining the above two points, it can be concluded that enhancing math self-efficacy is one of the effective ways to improve students’ mathematical literacy.

(4) Differences in key influences affecting math literacy and the extent of their contribution across individuals.

After a detailed analysis of the factors affecting the mathematics literacy of individual students as well as multiple students, it was found that, first, there are significant differences in the key influencing factors between students (see Figure 8 and Figure 9). Second, the same factor may make diametrically opposed contributions to the mathematics grades of different students (see Figure 8, Figure 9 and Figure 10). For instance, math self-efficacy (MATHEFF) may positively contribute to the model output of student A (top-performing student), while it shows a negative connection to student B (low-performing student). Third, the same factor contributes differently to the mathematics scores of different students. For example, support or feedback via ICT (ICTFEED) improves the mathematics scores of student A and student B by 8.01 and 8.34 points, respectively, from the average score of all samples. Meanwhile, the above results can provide valuable data support for personalized education based on artificial intelligence technology (Cao et al. 2024).

## 5. Conclusions and Limitations

This paper employs machine learning and the SHAP method to analyze 34,968 samples and 151 features from PISA 2022 in Hong Kong, Macao, Taipei, Singapore, Japan, and Korea, aiming to identify the key factors affecting middle-school students’ literacy in mathematics. It has several advantages. First, machine learning techniques in the field of education primarily focus on the prediction ability of the model and pay less attention to its explanatory nature. The article applies the SHAP method to explain the model output by calculating the extent to which each variable contributes to the forecasts. Second, important factors affecting mathematical literacy performance are identified by considering many features to obtain more comprehensive and insightful analysis results. Third, the global model explanation of the SHAP method can help provide specific and actionable ideas for improving the educational system in terms of enhancing students’ performance in mathematical literacy. Fourth, the dominant factors affecting the mathematical literacy of a specific individual, as revealed by the local model explanation, can provide a valuable database for the implementation of personalized instruction in the context of AI-assisted education.

There are 4 conclusions that can be obtained. First, the XGBoost model is considered the best regression algorithm for predicting mathematics literacy. Second, the results of the global model explanation show that the key factors affecting the mathematical literacy performance of all students include 15 variables, such as mathematical self-efficacy (MATHEFF, MATHEF21), expected occupation status (BSMJ), leisure activities with the help of ICT (ICTWKEND) and so on. Among them, mathematics self-efficacy (MATHEFF, MATHEF21), expected occupational status (BSMJ), family’s economic, social, and cultural status (ESCS), use of ICT for learning activities outside the classroom (ICTOUT), number of mathematics teachers in the school (TOTMATH), grade level (GRADE), and gender (ST004D01T) have a positive impact. Conversely, recreational activities through ICT (ICTWKEND), frequency of participation in sports (EXERPRAC), math anxiety (ANXMAT), disruptive behaviors such as truancy and alcohol abuse (STUBEHA), use of ICT to receive support and feedback from teachers and peers (ICTFEED), the ability grouping for mathematics classes (ABGMATH), and cognitive activation to encourage students to do mathematical thinking (COGACMCO) show a negative relationship. Third, math self-efficacy has the most significant effect on students’ math literacy. Fourth, important influences are not identical across individuals; the same factor may have positive or negative effects on various students and does not affect different students’ mathematical literacy to the same extent.

This study captures many samples and features, as well as the complex relationships between them through machine learning, and comprehends these relationships with the aid of SHAP methods to more profoundly and comprehensively reveal the important factors affecting mathematical literacy. In addition, the results of this kind of research have some insights into enhancing students’ mathematical literacy.

First, mathematical self-efficacy (MATHEFF, MATHEF21) plays a positive role in mathematical literacy and ranks first in terms of the degree of its influence. Therefore, teachers should pay attention to cultivating students’ self-efficacy in their daily teaching. For example, setting up reasonable problems and scenarios to help students gain a successful experience and improve their self-efficacy.

Second, the expected occupation status (BSMJ) is just after MATHEFF in terms of its importance to mathematical literacy and plays a positive role. In teachers’ instruction and extracurricular activities, emphasis should be placed on stimulating students’ intrinsic motivation to learn and raising the level of expected occupation.

Third, the frequency of ICT activity-weekend (ICTWKEND), exercising or practicing a sport before or after school (EXERPRAC), and mathematics anxiety (ANXMAT) have a negative effect on mathematics literacy. On the one hand, the larger the value of ICTWKEND and EXERPRAC, the worse the literacy. The possible reason is that students spend too much time on online networks or outside the classroom engaging in activities that are not related to academics. The school, parents, and teachers should rationalize the students’ time for study, rest, and recreation and help students build a healthy view of time. On the other hand, the larger the value of math anxiety, the worse the performance of mathematics literacy. Therefore, schools, parents, and teachers should view students’ academic scores correctly to avoid causing unnecessary anxiety to students. Moreover, some methods to reduce math anxiety should be taught to help students relieve math anxiety.

Fourth, the results of the SHAP local model explanation show that there are some differences in the key influence variables and their degree of influence across individuals. For this reason, when conditions permit, recommendations for individuals can be given based on the key factors it provides for influencing a specific individual and combined with other AI techniques to truly personalize education and teaching.

There are also some limitations. Regarding the data, first, the PISA dataset is derived from subjective responses from students, parents, school administrators, and teachers, which may affect the objectivity of the data. Second, the PISA data are characterized by multiple levels of nesting. However, machine learning neglects the interaction of student-level data with school-level data. Once again, the article mainly focuses on data from six East Asian education systems and only examines the prediction performance of the model on the U.S. and Spanish PISA 2022 datasets. In the future, we can consider incorporating data from more countries (regions) as well as from different years to test the prediction ability of the model. Finally, the data used in the SHAP partial dependence plot, such as MATHEFF, MATHEF21, and ANXMAT, are derived variables based on weighted likelihood estimates (WLE) and normalized to [0, 1]. This means that they do not directly reflect the specific impact of the raw data for each feature on math literacy. Regarding model selection, the study only compares the prediction ability of five machine learning methods, such as Support Vector Machine and XGBoost. Future research can consider introducing deep learning techniques to improve prediction accuracy. As for the model explanation, the SHAP method did not fully consider the causal relationship between features. Future research can try to adopt a model explanation method that considers the causal relationship between features to obtain a deeper understanding.

## Figures and Tables

**Table 1:** Set of hyperparameters for different methods.

Models	Hyperparameter Combinations	Optimal Hyperparameter Combination
MLR	default	default
SVR	‘C’: [0.01, 0.1, 1, 10, 100], ‘gamma’: [1, 0.1, 0.01, 0.001, 0.0001], ‘kernel’: [‘rbf’]	C = 100, gamma = 0.01, kernel = ‘rbf’
DT	‘max_features’: [‘sqrt’, ‘log2’, ‘None’], ‘max_depth’: [5, 6, 7, 8, 9, 10, 11, 12, 15, 20, 30], ‘criterion’: [‘squared_error’]	criterion = ‘squared_error’, max_depth = 8, max_features = ‘sqrt’
RF	‘n_estimators’: [100, 200, 300, 500], ‘criterion’: [‘squared_error’], ‘max_features’: [‘sqrt’, ‘log2’, ‘None’], ‘max_depth’: [6, 7, 8, 9, 10, 11, 12, 15, 20, 30, 40, 50]	max_features = ‘sqrt’, n_estimators = 500, max_depth = 30
XGBoost	‘learning_rate’: [0.01, 0.015, 0.025, 0.05, 0.1, 0.01, 0.015, 0.025, 0.05, 0.1], ‘gamma’: [0.05, 0.1, 0.3, 0.5, 0.7, 0.9, 1], ‘reg_alpha’: [0, 0.01, 0.1, 1], ‘reg_lambda’: [0, 0.1, 0.5, 1], ‘max_depth’: [3, 5, 6, 7, 9, 12, 15, 17, 25], ‘min_child_weight’: [1, 3, 5, 7], ‘subsample’: [0.6, 0.7, 0.8, 0.9, 1], ‘colsample_bytree’: [0.6, 0.7, 0.8, 0.9,1], ‘objective’: [“reg: squarederror”]	learning_rate = 0.1, gamma = 0.05, reg_alpha = 0.01, reg_lambda = 0.5, max_depth = 7, min_child_weight = 3, subsample = 1, colsample_bytree = 0.6, objective = “reg: squarederror”

**Table 2:** Assessment metrics of optimal parameter models for different models.

Models	MSE	RMSE	MAE	MAPE	$R^{2}$	PCCs
MLR	5457.59	73.88	58.2	11.57%	0.49	0.70
SVR	4560.49	67.53	52.93	10.51%	0.58	0.76
DT	7279.3	85.32	67.85	13.50%	0.32	0.57
RF	4926.58	70.19	55.42	11.08%	0.54	0.75
XGBoost	4344.98	65.92	51.72	10.21%	0.60	0.77

**Table 3:** Prediction capability of XGBoost with a different number of features.

Numbers of Features	MSE	RMSE	MAE	$R^{2}$	MAPE	PCCs
5	5791.34	76.10	60.08	0.46	11.91%	0.68
10	5155.85	71.80	56.41	0.52	11.18%	0.72
15	4969.04	70.49	55.34	0.54	10.95%	0.73
20	4807.67	69.34	54.46	0.55	10.80%	0.74
30	4545.81	67.42	53.02	0.58	10.49%	0.76
40	4501.69	67.09	52.70	0.58	10.41%	0.76
50	4386.68	66.23	52.03	0.59	10.25%	0.77
60	4346.37	65.93	51.98	0.60	10.26%	0.77
72	4342.73	65.90	51.87	0.60	10.25%	0.77

## Data Availability

The datasets used and/or analyzed during the current study are available from the corresponding author upon reasonable request.

## Supplementary Materials

The following supporting information can be downloaded at https://www.mdpi.com/article/10.3390/jintelligence12100093/s1, Figure S1: Comparison of Hong Kong math literacy performance scores estimated by the XGBoost algorithm with the actual results; Figure S2: Comparison of Korea math literacy performance scores estimated by the XGBoost algorithm with actual results; Figure S3: Comparison of U.S. math literacy performance scores estimated by the XGBoost algorithm with actual results; Figure S4: Comparison of Spain math literacy performance scores estimated by the XGBoost algorithm with actual results; Table S1. 72 variables used for model training; Table S2: Prediction accuracy of XGBoost on PISA math literacy performance in Hong Kong, Korea, the United States and Spain.
