# Supplementary material for: Applying Machine Learning and SHAP Method to Identify Key Influences on Middle-School Students’ Mathematics Literacy Performance
Source: J Intell. 2024 Sep 26;12(10):93. doi: 10.3390/jintelligence12100093 (PMC11508920; doi:10.3390/jintelligence12100093)
Supplement: Supplementary file 1 [file jintelligence-12-00093-s001.zip › jintelligence-3093089-supplementary.pdf]

Supplementary Material

1 Supplementary Figures

Figure S1: Comparison of Hong Kong math literacy performance scores estimated by the XGBoost algorithm with the actual results.

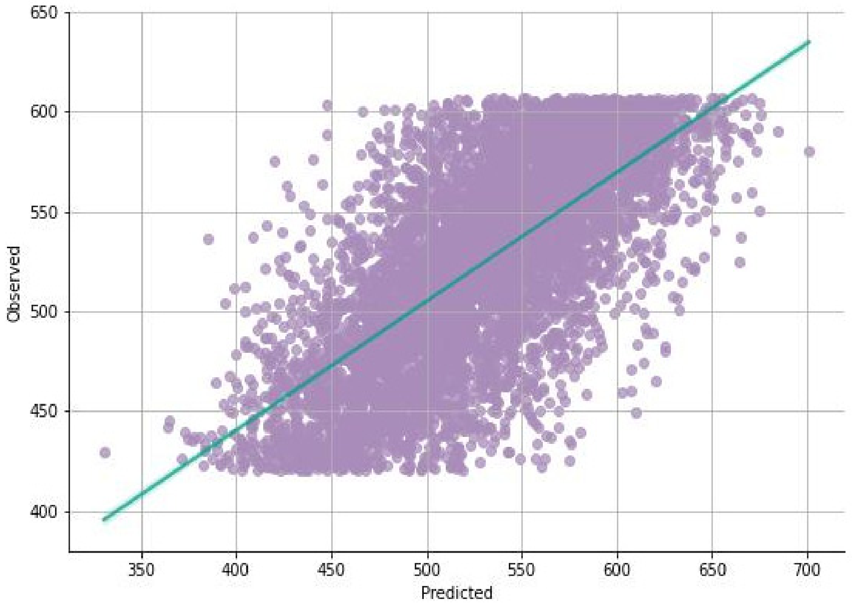

Figure S2: Comparison of Korea math literacy performance scores estimated by the XGBoost algorithm with actual results.

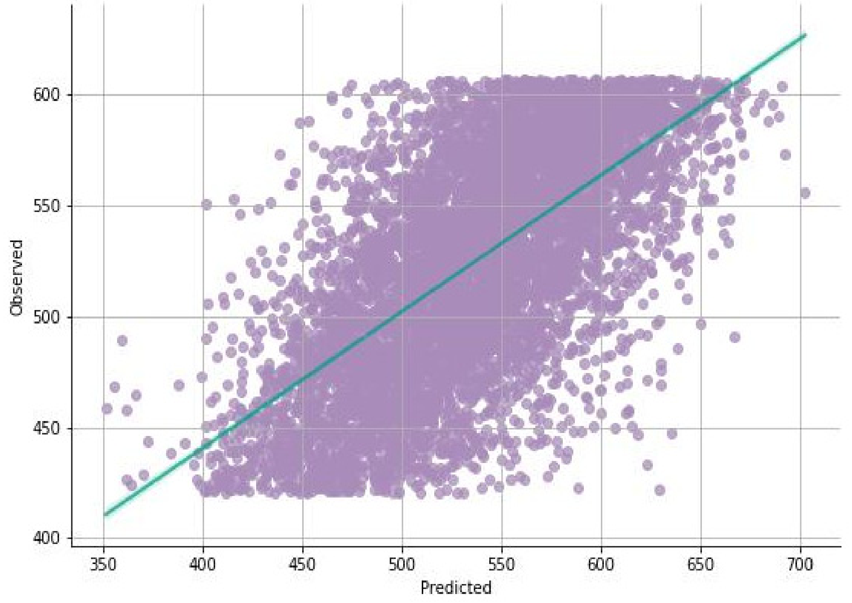

Figure S3: Comparison of U.S. math literacy performance scores estimated by the XGBoost algorithm with actual results.

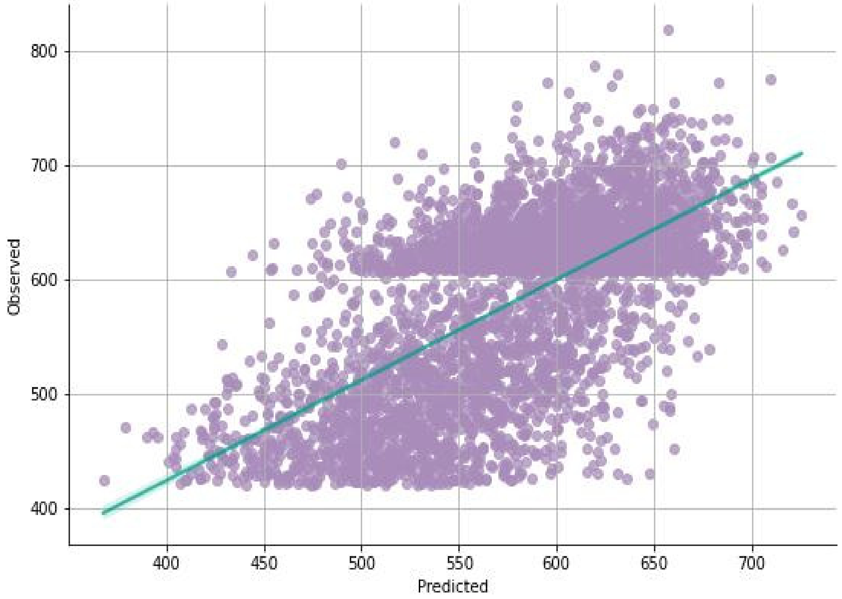

Figure S4: Comparison of Spain math literacy performance scores estimated by the XGBoost algorithm with actual results.

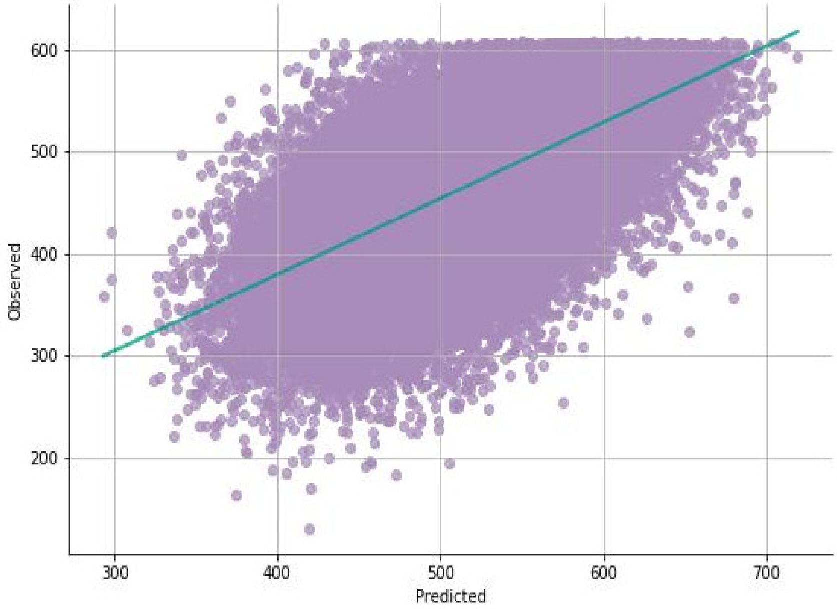

2 Supplementary Tables

Table S2. 72 variables used for model training.

Variables	Variable Description Information
ABGMATH	Ability grouping for mathematics classes
ANXMAT	Mathematics Anxiety (WLE)
BELONG	Sense of belonging (WLE)
BFMJ2	Father's occupational status (ISEI) based on 4-digit human coded ISCO
BMMJ1	Mother's occupational status (ISEI) based on 4-digit human coded ISCO
BSMJ	Expected occupation status (free response)- 4 digits
BULLIED	Being bullied (WLE)
CLSIZE	Class size (test language class)
COGACMCO	Cognitive activation in mathematics: Encourage mathematical thinking Version B (WLE)
COGACRCO	Cognitive activation in mathematics: Foster reasoning Version B (WLE)
DISCLIM	Disciplinary climate in mathematics (WLE)
DURECEC	Duration in early childhood education and care
ENCOURPG	School encouragement of parent or guardian involvement (WLE)
ESCS	Index of economic, social and cultural status
EXERPRAC	Exercise or practice a sport before or after school
EXPO21ST	Exposure to Mathematical Reasoning and 21st century mathematics tasks (WLE)
EXPOFA	Exposure to Formal and Applied Mathematics Tasks (WLE)
FAMSUP	Family support (WLE)
FISCED	Father's level of education (ISCED)
GRADE	Grade compared to modal grade in country
ICTEFFIC	Self-efficacy in digital competencies (WLE)
ICTENQ	Use of ICT in enquiry-based learning activities (WLE)
ICTFEED	Support or feedback via ICT (WLE)
ICTHOME	ICT availability outside of school (WLE)
ICTINFO	Students' practices regarding online information (WLE)
ICTOUT	Use of ICT for school activities outside of the classroom (WLE)
ICTQUAL	Quality of access to ICT (WLE)
ICTREG	Views of regulated ICT use in school (WLE)
ICTSCH	ICT availability at school (WLE)
ICTSUBJ	Subject-related ICT Use During Lessons (WLE)
ICTWKEND	Frequency of ICT activity (weekend) (WLE)
MACTIV	Mathematics-related extra-curricular activities at school
MATHEASE	Perception of Mathematics as easier than other subjects

MATHEF21	Mathematics self-efficacy: mathematical reasoning and 21st century skills (WLE)
MATHEFF	Mathematics self-efficacy: formal and applied mathematics - response options reversed in 2022 (WLE)
MATHEXC	Mathematics extension courses offered at school
MATHMOT	Motivation to do well in mathematics
MATHPERS	Effort and Persistence in Mathematics (WLE)
MATHPREF	Preference of Math over other core subjects
MTTRAIN	Mathematics teacher training (WLE)
PROADMIN	Proportion of school administrative personnel
PROATCE	Proportion of all teachers fully certified
PROMGMT	Proportion of school management personnel
PROOSTAF	Proportion of other non-teaching staff
PROPMATH	Proportion of mathematics teachers at school
PROPSUPP	Proportion of personnel for pedagogical support
RATCMP1	Availability of computers
RATCMP2	Computers connected to the Internet
RATTAB	Availability of tablet devices
SC001Q01TA	Which of the following definitions best describes the community in which your school is located?
SC011Q01TA	Which of the following statements best describes the schooling available to students in your location?
SC042Q01TA	School's policy for [national modal grade for 15-year-olds] students: Students are grouped by ability into different classes.
SC042Q02TA	School's policy for [national modal grade for 15-year-olds] students: Students are grouped by ability within their classes.
SC187Q03WA	Which describes school's math classes: Students are grouped by ability within their mathematics classes.
SCHAUTO	School autonomy (WLE)
SCHSEL	School selectivity
SCHSIZE	School size (Sum)
SISCO	Clear idea about future job
SKIPPING	Skipping classes or days of school
SMRATIO	Student-mathematics teacher ratio
SRESPCUR	School responsibility for curriculum
SRESPRES	School responsibility for resources
ST004D01T	Student (Standardized) Gender
STRATIO	Student-teacher ratio
STUBEHA	Student-related factors affecting school climate (WLE)

TARDYSD	Arriving late for school stricter definition
TCHPART	Teacher participation (WLE)
TEACHSUP	Mathematics Teacher Support (WLE)
TEAFDBK	Feedback to teachers (WLE)
TOTAT	Total number of all teachers at school (Sum)
TOTMATH	Total number of mathematics teachers at school (Sum)
TOTSTAFF	Total number of non-teaching staff at school

Table S2: Prediction accuracy of XGBoost on PISA math literacy performance in Hong Kong, Korea, the United States and Spain.

Region	MSE	RMSE	MAE	MAPE	PCCs
Hong Kong	1820.50	42.67	33.67	6.53%	0.69
Korea	2075.56	45.56	35.86	6.99%	0.66
USA	3795.71	61.61	49.37	8.85%	0.65
Spain	6595.17	81.21	65.18	15.57%	0.61

MSE: Mean Square Error; RMSE: Root Mean Square Error; MAE: Mean Absolute Error; MAPE: Mean Absolute Percentage Error; R^2: Coefficient of Determination; PCCs: Pearson Correlation Coefficients.
